# Supplementary material for: Social norms and maternal health information-seeking behavior among adolescent girls: A qualitative study in a slum of Bangladesh
Source: PLoS One. 2024 Dec 6;19(12):e0315002. doi: 10.1371/journal.pone.0315002 (PMC11623481; doi:10.1371/journal.pone.0315002)
Supplement: Attachment — Submitted filename: Response to Reviewers_140624.docx [file pone.0315002.s002.docx]

June 14, 2024

Sanzida Akhter, PhD

Academic Editor

PLOS ONE

Manuscript Number: PONE-D-23-34519

Title: A Qualitative Study on the Maternal Health Information-Seeking Behavior among Adolescent Girls of Reproductive Age in a Slum of Dhaka, Bangladesh

Dear Editor,

Thank you for allowing us to resubmit the revised manuscript. We appreciate the time and effort that you and the reviewers have dedicated to providing us with your valuable feedback on our manuscript. We are truly grateful to the reviewers for their insightful comments on our paper. It was a great learning for us. We believe these suggestions will help improve the quality of the manuscript. Therefore, we have incorporated changes to reflect all the suggestions provided by the reviewers. We have used track changes option within the manuscript to highlight the changes.

In addition, this is to inform you that as some of our affiliation has changed by this time, we have changed that in the portal and the title page.

Here is the point by point responses to the reviewers’ comments and concerns:

**Response to Reviewer #1:**

**Reviewer #1: Review of study entitled “A Qualitative Study on the Maternal Health Information-Seeking Behavior among Adolescent Girls of Reproductive Age in a Slum of Dhaka, Bangladesh”

Reviewers Comment**

**Reviewer point #1: This is a crucial topic in addressing the unmet needs of adolescents Maternal Health Information-Seeking Behavior among Adolescent Girls of Reproductive Age in a Slum in Dhaka, Bangladesh but the findings of the study were not elaborate enough even though exploratory design was employed.**

**Author response #1:** *Thanks for your in-depth feedback. We have tried to incorporate elaboration for your reference. As this paper was a maiden paper from the team, we faced some struggles earlier in coordinating and collaborating.*

*Major changes in this version:*

1. *Theoretical model is added*
2. *Methods are re-written*
3. *Grammar is revised*
4. *Results are re-written in elaborative manner*
5. *Structure is revised*

**Reviewer point #2. Language: Authors need to improve on the grammar of the manuscript. Sometimes authors use ‘previous studies’ but refer to only one study, see introduction, methodology sections etcetera. Also, authors use certain terms that are not familiar in the Englush language.**

**Author response #2:** We agree on this comment. The whole paper has been reviewed and find grammatical and logical flaws. The concern of “**Sometimes authors use ‘previous studies’ but refer to only one study”** were addressed.

**Reviewer point #3. The title: “A Qualitative Study on the Maternal Health Information-Seeking Behavior among Adolescent Girls of Reproductive Age in a Slum of Dhaka, Bangladesh”. This is confusing, what does adolescent girls of reproductive age mean?**

**Author response #3:** *We put the words ‘adolescent girls of reproductive age’ by considering the mostly known definition of adolescents, who are from 10-19 years, and the reproductive age of a women, which is 15-49 years. In such sense, adolescent girls of reproductive age refer to the girl who are between 15 and 19 years old. We set this explanation in fourth sentence of* ***Study Design*** *in* ***Methodology*** *section.*

*However, we have changed our research title from* ***A Qualitative Study on the Maternal Health Information-Seeking Behavior among Adolescent Girls of Reproductive Age in a Slum of Dhaka, Bangladesh*** *to* ***Social norms and maternal health information-seeking behavior among adolescent girls of reproductive age: A qualitative study in a slum of Bangladesh.***

**Reviewer point #4. Abstract: Authors do not indicate which category of participants the IDIs and FGDs were conducted among. The results section lacks completeness and a logical flow as well as how and why the source of information is not considered a problem.**

**Author response #4:** *Categories are mentioned in Abstract’s Method section. Also, we have included theoretical framework as the foundation for the study's conduct and design.*

***Edited version:4***

**Methods**: Adopting an explorative qualitative research approach, we collected data from purposively selected married and unmarried adolescent girls (15-19 years old) of different occupation by implying 12 in-depth interviews (IDIs), two Focus Group Discussions (FGDs) with same categories employed for IDIs, and two Key Informant Interviews (KIIs) with a traditional birth attendant and a local pharmacist. Furthermore, the data were subjected to thematic analysis. Care’s Social Norms Analysis Plot (SNAP) framework were undertaken as interpretative tool for data that was emerging rather than serving as the foundation for the study's conduct and design. Thematic analysis were followed to analyze primary data.

*The* ***Result*** *section of* ***Abstract*** *is re-written by addressing reviewer’s comment.*

**Results:** Majority reported that adolescent girls need professional healthcare providers in their area who would work according to their work schedule as most of the girls are engaged in income generating work for about 9-11 hours, and the scope of work (daily wagers) hardly supports ‘leave with pay’. In the present context, the most of the adolescent girls rely on elder female family members, traditional birth attendants, and local pharmacists for maternal health information. These unverified sources prescribe treatment such as medicine, food, and herbal products without formal expertise which causes life risks for adolescent mothers. Additionally, existing social norms and stigma have a bigger impact on the maternal health information seeking of adolescent girls.

**Reviewer point #5. The conclusion in the abstract appears more like a recommendation. The authors can review the abstract conclusion.**

**Author response #5:** *The Conclusion section is re-written by addressing reviewer’s comment.*

**Conclusion**: The study offers a different narrative in the discussion of maternal health information seeking behavior, specifically how social norms and stigma are preventing slum adolescents to get their desired health information. The results might be useful for informing policy and program designing to ensure better health outcome for marginalized adolescents.

**Reviewer point #6. Introduction: The introduction is not focused and lacks a logical flow. Authors need to be more focused.**

**Author response #6:** We have tried to rewrite the **Introduction** section, accordingly.

# INTRODUCTION

Maternal health is a worldwide issue, even though it was reduced by 38% from 451,000 in the year 2000 to 295,000 deaths in 2017. This is a particular problem for developing countries where 94% of maternal deaths occur (1). In Bangladesh, reducing maternal mortality is a national priority, supported by policies like the Bangladesh National Health Policy 2011 (2); as long as with the Bangladesh National Strategy for Maternal Health 2019-2030 (3). The issues of maternal health are in the attempts to reduce all the factors that negatively affect the physical condition and social position of women and mothers. At the present moment, the number of the mortality rates had reduced in comparison to 2000 when it was 434 per 100,000 live births, and in 2017, it was 173, whereas the current goal for 2030 is to make it lower than 70 (1).

Bangladesh has one of the highest adolescent fertility rates in the Asia Pacific, with 128 births per 1000 girls aged 15 to 19 (4). Adolescent girls are married off 3-4 years earlier than the legal marriage age of 18. Married adolescent girls go through unavoidable social and family pressure to get pregnant soon after marriage as proof of their fertility (5). Their incapacity to obtain family planning (FP) and reproductive health (RH) services encourages early childbirth and marriage (6). In contrast to rural women, women living in urban regions are more likely to have made four or more antenatal visits (59% vs. 43%) (7). Again, the health-seeking behavior of people living in cities varies. Evidence illustrates that 20% of teenage mothers gave birth unintendedly (5), and unwanted pregnancies are more than twice as common among married adolescent girls in Bangladesh's slums as in non-slum areas (6). Previous study demonstrates that Bangladeshi slum had a maternal mortality rate of 294 deaths per 1000,000 live births and a neonatal mortality rate of 43 deaths per 1,000 live births (8).

Access to timely and relevant maternal health information is crucial for informed decision-making and reducing maternal morbidity and mortality. Accurate maternal health information promotes positive health behaviors and better maternal health outcomes (9). Delays in seeking appropriate care during pregnancy significantly increase maternal risks. While some studies have addressed maternal and neonatal mortality in slums, there is a notable gap in research on the health information-seeking behavior of adolescent girls in Bangladesh's urban slums. This is critical, given that slums host over 5.7 million people, approximately 3.8% of the national population, most of whom are migrants seeking better economic opportunities (10). Addressing the maternal health information needs of this marginalized group is essential for improving maternal health outcomes in Bangladesh.

Care’s Social Norms Analysis Plot (SNAP) Framework was developed to measure the nature of specific social norms and their influence and offers a useful framework to examine the initial reactions to a social norms activity. Empirical expectations, normative expectations, sanctions, sensitivity to sanctions, and exceptions are five key constructs to understand one’s act or decision-making (11). Although studies exist on overall unexpected pregnancy and contraceptive use among married adolescent girls in Bangladesh, data on particularly vulnerable groups (as our study examines both married and unmarried adolescents from an urban slum) of adolescents is scant. In addition, to our best knowledge, there is no research done yet to understand the maternal health care information seeking in social norm perspective. Therefore, we aim to explore the maternal health information-seeking behavior of adolescent girls of reproductive age in a slum through the lens of social norms. The primary goals of this study were to identify adolescent girls' maternal health information needs during their reproductive years and to learn about how social norms working as the obstacles they face when seeking information related to maternal health.

**Reviewer point #7. The methods section is scanty. Consider using the Consolidated criteria for reporting qualitative research (COREQ) to report on the missing aspects.**

**Author response #7:** *Thanks. We have incorporated Consolidated criteria for reporting qualitative research (COREQ) to report on the missing aspects. The overall method section is re-written and currently we have* ***Study Design****,* ***Study Setting, Sampling and Recruitment, and Data Collection Procedure, Data Analysis, Ethical Consideration and Ethical Clearance*** *sub-section.*

**Reviewer point #7 (a). Also, the design is mentioned twice**

**Author response #7 (a):** *Thanks. We have addressed the concern.*

**Reviewer point #7 (b). Authors failed to explain the type of purposive sampling technique that was used**

**Author response #7 (b):** *The explanation is given.*

## **Sampling and Recruitment**

The study used purposive sampling method to recruit study participants as per convenience due to the variety of occupational engagement of the study population. To ensure confidentiality when discussing delicate subjects, participants were assigned with same-sex data collectors. The data collectors were trained anthropologists and geographer. The field researchers received three days of training from the lead investigators, with a focus on sensitive research involving human subjects and notably adolescent subjects conducted in an ethical manner. To maximize comprehension and enable correct response documentation, the IDI, FGD and KII guides with vignettes were tested, revised, and collaboratively translated into Bengali and back into English.

**Reviewer point #7 (c). Authors fail to report on how participants were recruited, the focus of the IDI, KII and FGD guides, the pharmacists are missing from the table**

**Author response #7 (c):** *We have added the ways we followed to recruit participants. Please, see the Methodology section*

**Reviewer point #7 (d). The authors fail to explain the reasons for choosing the study design and why pharmacists were included. Generally, there is poor or lack of justification for decisions made in the methodology section**

**Author response #7 (d):** *In the edited version of the paper, we have explained the justification. Also, we added why we recruit the KIIs.*

## **Study Design**

We undertook an exploratory qualitative method, a widely used methodological approach, especially within practice disciplines (12), to have a deeper understanding of the maternal health information-seeking behavior among adolescent girls of reproductive age in a slum of Dhaka, Bangladesh. We used the terms- adolescent girls of reproductive age, by considering the mostly known definition of adolescents, who are from 10-19 years, and the reproductive age of a women, which is 15-49 years, according to WHO (13, 14). In such sense, adolescent girls of reproductive age refer to the girl who are between 15 and 19 years old. This approach was selected for this study due to its nature of nature of investigating research objectives by exploratory questions (15). The lack of in-depth research on maternal healthcare access among this age group, living in slums, led us to a need for an in-depth exploratory study. For this, we selected in-depth interview (IDI), focus group discussion (FGD) and key informant interview (KII) (Table 1), to provide a data source for triangulation. IDI participants provided subjective viewpoints and FGD participants helped us to understand the community perspective. Participants of KII facilitated to get objective viewpoints of the data collected by other methods. We conducted IDIs with six married adolescent girls (MAG) and six unmarried adolescent girls (UAG) and two FGDs with MFAG and UFAG. Among two KII participants, one was local traditional birth attendant (TBA) and the other was a pharmacist by profession. TBA was recruited as majority of the study participants feel comfortable to visit her. On the other hand, pharmacist is the key person to the slum to take medicine. Participants consider them as doctor and take medicine from them without prescription, rather just telling health problems. Despite UAGs’ and MAGs’ access to *Kabiraj* (faith healer), we could not interview him as he was outside of the slum due to personal reasons. Furthermore, we approached formal health service providers whose facility was closer to the slum, however, as he reported that he received any adolescent girl from the studied slum as patient, we did not consider them as KII.

**Reviewer point #8. The narration in the results section sometimes appears to be more of commentary than reporting qualitative results from study participants eg first paragraph on page 8.**

**Author response #8:** *Thanks. We have rewritten the paragraph.*

**Reviewer point #8 (a). I could not comprehend table 4**

**Author response #8 (a):** *We agree with you. The table is removed. We tried to illustrate the consequences of unintended pregnancy for the lack of knowledge, earlier.*

**Reviewer point #8 (b). The authors should improve on the structure. Sometimes a topic is introduced without any warning or when it is not in relation to information in that section for instance the narration on fistula, should have a separate section, it does not fit well, where it is currently.**

**Author response #8 (b)** *We focused on structure in the revised version.*

**Reviewer point #**9. **This is an important topic that extends beyond the study area, but the literature used in the discussion section is very scanty. The authors fail to make their paper relevant to the broader society by not engaging extensively in literature pertaining to other parts of the world.**

**Author response #9:** *We have re-written the discussion by relating other scholarly research work all over the word*

**Reviewer point #10. The recommendation is not adequate and not targeted to specific policymakers and other stakeholders**

**Author response #10:** *Please, find the following edited version:*

## **Policy recommendations and future research**

Findings suggests three core recommendations. First, to organize and arrange the health service for adolescents by considering their occupational engagement. More specifically, before providing health service, it is essential to ensure the proper information is disseminated properly. If not, the overall health problem solving goal would be facing difficulties. Second, only targeting the adolescents for awareness session is not enough. As the husbands and in laws members are the key decision makers, it must be ensured that these people are well-aware of the risks of lack of maternal health care information. Third, programs targeting to reduce maternal mortality rate, adolescent sexual and reproductive health and rights (SRHR) should focus more on social norms and stigma as these are embedded in-depth into our society and culture.

For further research, in our opinion, more studies should be done on context-specific social norms. In Bangladesh context, there is a huge gap of research on social norms regarding maternal health information seeking behavior and other SRHR issues using social norm’s framework.

**Reviewer point #11. The recommendation should be separated from the conclusion**

**Author response #11:** *We have separated Recommendation from Conclusion. Please, find the previous response.*

**Reviewer point #12. The study did not talk about the available health facilities in Sattola and its surrounding**

**Author response #12:** *Right. We have mentioned the available health facilities in and surrounding Sattola slum.*

## **Study Setting**

The study was done on Sattola, a slum of Dhaka city, Bangladesh because of its highly dense population and urban slums are well-known for the highest rate of teenage mother. From our observation, the area is shoddy, with below standard houses built by less expense; resultant of this is inadequate sanitation, deficiencies in access to basic services such as health insurance. The people of Sattola are not only battling with high rates of adolescent pregnancy but they also subject to a paramount of material constraints. No particular NGO is found working on adolescent maternal health care during the fieldwork. However, there are some treatment facilities which is available at the International Centre for Diarrheal Disease Research, Bangladesh and Universal Medical College and Hospital which provide some diagnosis service related to maternal health. These medical facilities are situated within a 2-3 kilometer radius of most of the households.

**Reviewer point #13. To obtain a more accurate picture of the situation in Sattola Slum, it would be great if health professionals were also included in the study participants to know their engagement efforts and barriers to reaching out to adolescent females as well as unmarried and married adolescent males.**

**Author response #13**: *We had the same thought earlier. However, as the adolescent girls of the slum hardly visit any health professionals due to lack of proper time, social norms and so forth, we considered that health professional would not be the appropriate person as participant. Instead, as per adolescents’ current practice of seeking maternal healthcare information from TBA and Pharmacist, we conducted KII from these persons.*

**Response to Reviewer #2:**

**Reviewer point #1:** Overall, the authors tried to fulfil the objectives of the study. However, the authors could not address gender issues properly when they were analysing things. At some point, the gender lens is missing from this article. They mentioned many things. However, this was not properly aligned with gender terms like gender needs (Practical and strategic gender needs), and social stigma. Also, they mentioned different kinds of identities in demographic profiles as well. However, the analysis part could not give the proper justice to these different kinds of intersectional identities. They should have provided a deeper analysis of how married women are not free to make any decision according to their will, and why unmarried adolescent girls are not willing to talk. More details and a thorough analysis of this part with a combination of primary and secondary data would be expected.

**Author response #1:** *Thanks very much for your feedback. We have tried to address your comment. Please, find the edited Discussion section:*

# Discussion

The study intended to explore the health information-seeking behavior of adolescent girls of reproductive age in the slum context of Bangladesh. It is clear from the data that the necessities for accurate maternal health information are not being met in the studied slums, which are consequently hampered by constraints like social norms and stigma, a lack of education and awareness, the timing of service provision, and so forth (Fig 1). The findings of this study highlight several critical aspects of maternal health information needs among adolescent girls in urban slums, revealing new insights and differences from previous research, particularly regarding gender dynamics and intersectional identities.

The unfamiliarity of participants, especially first-time adolescent mothers, with early pregnancy symptoms and associated risks underscores the low awareness about maternal health in the studied slum areas. This is consistent with wider studies in sub-Saharan Africa and South Asia and globally where a multiconceptual background to pregnancy health and danger signs is associated with increased maternal morbidity and mortality (17). Like with the adolescents in our study, they sourced maternal health information mainly from unverified sources, which has also been found in rural India and Nigeria due to lack of access to credible health information (18-20). These parallels suggest that low awareness and reliance on unverified sources are common challenges in under-resourced settings.

Our study results are in contrast with other research works especially of Kamal et al (2015) and Haider et al (2023) (21-22). Evidence was also reported from similar settings to include Schuler et al. (2006) in Bangladesh where logistical barriers such as time and service inadequacy were experienced were main causes to inquire about non-certified knowledge (23). On the contrary, our findings suggest that it is the time poverty and failure of mobilization within that proposed to be driving youth to questionable sources. This disparity demonstrates the need of context-appropriate interventions to remove barriers to access of information regarding maternal health.

The research findings of this study confirms with the study by Zia et al. (2021) emphasizes that stigma results in significant underreporting of unsafe and clandestine abortions, especially in sub-Saharan Africa (24). Such shame and stigma together result in an important psychosocial burden on the lives of young women experiencing abortion, and can justify the moral, and frank ideological dilemmas encountered by them. This study outlines specific slum characteristics related to social dynamics. Findings say that UAGs in the slums are hesitant to disclose abortion because they further violent social repercussions. Identification through a successful abortion would expose a UAG to a lifetime of societal censure and escalation by rendering her unmarriageable and putting her and her family at risk for public ostracism and violence. It corresponds with the wider impression of stigma-related difficulties highlighted in previous literature as well (25). Additionally, our study suggests that interventions to reduce abortion stigma must be tailored to the complex social norms and consequences of abortion stigma for women living in predominantly slums, which highlights a unique area for future research and intervention development (24-25).

The research also revealed a profound unawareness of family planning and obstetric fistula among adolescent girls. These findings are consistent with a body of evidence from Ethiopia and Kenya which shows how entrenched cultural beliefs and poor sexual and reproductive health education perpetuate misconceptions about reproductive health (26, 27). Although we already know that previous research has shown misinformation about such issues (28), our data shows how such knowledge gaps are exacerbated by social stigma and certain cultural beliefs of witchcraft as a cause of obstetric fistula. Additionally, this ignorance getting in the way of teenagers being able to use the health service when they are there to be used, as well as continuing the problem practices and beliefs. The results seem to call for the development of more specific educational programs on family planning, which should aim at responding to women's practical and strategic gender needs by disapproving unhelpful myths and providing reinforcing messages (29).

Although health-information seeking from informal sources-such as elder female relatives-is not only common in the studied slums but also in patriarchal societies around the world. The studies in Afghanistan and Nepal show, accompanied with the ego constructs and social norms, women have less mobility and less usage of the formal health services than men and the women often rely on the family members for their health information (30-31). Taken together, these findings suggest that community health workers and informal networks should be integrated into more formally directed health education programs to ensure accurate information is spread.

**Reviewer point #2: They interviewed a few numbers of IDI among unmarried girls. Therefore, it’s not okay to perceive that unmarried participants were not willing to share their abortion history. Probably, they did not have. As they mentioned, “Though KII participants clarified that they know of some cases of abortion by unmarried adolescent girls, no unmarried IDI participant was keen to share those pieces of information with us.” The authors need to explain this statement more. Why do they think like that? What kinds of stigma do they face from the society? Also, they need to back up their primary data providing secondary data as well. In the analysis section, primary data is not getting enough backup or supported by secondary data.**

**Author response #2:** You are absolutely right. We missed to mention about the social stigma described by the KIIs. We incorporated the findings in the result section. However, in our opinion, the number of the reported case of aborting babies by UAGs are very low. Moreover, there is a high possibility that the purposively selected participants are not included in those reported cases by KII. That’s why we did not find any data from the UAGs regarding.

*Edited:*

Though KII participants clarified that they know of some cases of abortion by UAGs, no unmarried IDI participant was keen to share those pieces of information with us. The major reasons of not reporting about abortion by the UAGs were existing social stigma. To illustrate, if people of the slum comes to know that any particular UAG has aborted child, she would never be able to get married in their slum. Moreover, her and her family’s dignity would be diminished.

*“In the slum, if any UAG is identified that she got pregnant, her life would be finished. If she has any younger sister, she would be treated badly and harassed by young boys of the slum. Overall, her family would be losing its dignity, fully.”* (KII, pharmacist, age 39)

**Reviewer point #3:** Without having the proper knowledge of sexual intercourse, they can become pregnant. At the same time, they can get STDs as well. However, this was not mentioned in this article. The authors could come up with a recommendation like providing door-to-door health services on weekends as well. The authors could have mentioned the discussion from the “Three Delay Model” and aligned it with their study. At some point, I felt it was necessary to talk with their family members like husband/partner to understand their conjugal understanding and decision-making process. It was also necessary to talk with parents or in-laws to focus on the stigmatized views of society.

**Author response #3:** *Thanks for suggesting the recommendation. As the study was focused on adolescent girls’ perception, we did not engage their parents. We just tried to be focused on adolescent’s viewpoint. This explanation is added to the sub-section entitled* ***Strengths and limitations.***

*Thanks for suggesting “Three Delay Model”. It is somehow related. However, we adopted* ***Care’s Social Norms Analysis Plot (SNAP) framework*** *as we tried to explore the existing social norms, in-depth. The revised version includes Care’s Social Norms Analysis Plot (SNAP) framework and interprets the data according to this framework. Because of this change, we have changed our research title from* ***A Qualitative Study on the Maternal Health Information-Seeking Behavior among Adolescent Girls of Reproductive Age in a Slum of Dhaka, Bangladesh*** *to* ***Social Norms and Maternal Health Information-Seeking Behavior among Adolescent Girls of Reproductive Age: A Qualitative Study in a Slum of Bangladesh***

*This is the basic framework:*

**Table 5: Component and response of social norms**

| Sl. | Components of Norm | Definition | Response from MAGs | Response from UAGs |
| --- | --- | --- | --- | --- |
| 1. | **Empirical Expectation** | What I think others do | 1. MAGs from richer families get the information from formal sources | 1. UAG do not seek maternal healthcare information |
| 2. | **Normative Expectation** | What I think others expect me to do (what I should do according to other) | 1. Elder female members are expected to ask for maternal health care information by MAGs  2. Girls should have baby immediately after marriage | 1. UAGs do not need to know about maternal health care information |
| 3. | **Sanction** | Expected reaction of other (to the behavior) – specifically others whose opinions matter to me. | 1. People would say ‘nashta’ (Person with bad characteristics) if not carry babies earlier | 1. People would term ‘besshya’ (prostitute) if anyone come to know that an UAG seek maternal healthcare information |
| 4. | **Sensitivity to Sanctions** | Do sanctions matter for behavior? If there is a negative reaction from others (negative saction), would the main character change their behavior in the future? | 1. Rich MAGs can visit formal information source  2. Poor MAGs cannot visit formal information source | 1. Neither rich nor poor UAGs can visit formal information source to seek these information |
| 5. | **Exceptions** | Under what conditions, it is okay to break the norm? | 1. If have rich family background  2. If have educated family background | 1. No exception is found |

**Reviewer point #4: In the discussion part, the authors tried to mention how this study is similar to other studies but it was also necessary to mention how this study is different from other studies, and what new pieces of knowledge or information are produced by this study. Is it bringing something new for us? Also, analysing the position and condition of these adolescent girls in a patriarchal setup like a slum area was not investigated properly. As this study is concerned with marginal women, it is necessary to have a gender lens to analyse the findings properly. How women were controlled, how their mobility was controlled, how they were bound to act according to imposed in-laws' decisions, and how these facts are shaping adolescents' maternal health information-seeking behaviour. These things should be discussed in the author's write-up. Overall, the theoretical and conceptual part was ignored in this study.**

**Author response #4:** *Yes. We totally agree with you. Please, find the updated Discussion section.*

**Reviewer point #5: Furthermore, the authors should be more careful about sentence formation. “The results of our study are different from those of earlier studies (18) which found that obstacles like long travel times to medical facilities and substandard services on maternal health from outreach programs force women to approach unauthorized providers to accumulate information in the slums, which are situated in a city.” It is suggested to avoid long lines like this as this is a little bit confusing for readers. Also, the conclusion should bring more information or recommendations from this study.**

**Author response #5:** *Thanks for your valuable feedback. We have revised the whole manuscript and re-written the larger sentences.*
