# Supplementary material for: Social norms and maternal health information-seeking behavior among adolescent girls: A qualitative study in a slum of Bangladesh
Source: PLoS One. 2024 Dec 6;19(12):e0315002. doi: 10.1371/journal.pone.0315002 (PMC11623481; doi:10.1371/journal.pone.0315002)
Supplement: Attachment — Submitted filename: Plos One Comments to the Author.docx [file pone.0315002.s003.docx]

**Manuscript ID**: PONE-D-23-34519R1

**Manuscript Title**: Social norms and maternal health information-seeking behavior among adolescent girls of reproductive age: A qualitative study in a slum of Bangladesh

Dear Md. Ashraful Haque,

Thank you for the opportunity to review this manuscript. This is an interesting study that offers some insights into the maternal health of adolescent girls in an urban setting in Bangladesh. The issue of social norms and maternal health-seeking behavior among adolescent girls is an increasingly important subject, especially in light of the current global health complexities. The links to maternal health information seeking and influences of social norms among adolescent girls everyday ways of activities are also important and could contribute to the growing literature around this subject, especially in the urban slum context who are mostly migrated from the rural areas. However, I agree that the qualitative methods and sample size appear appropriate except for the KIIs number. The manuscript may be publishable, but I have a number of considerable concerns that should be addressed.

1. The title could be revised. ‘Adolescent girls of reproductive age’ is likely unnecessary, as adolescence represents the onset of reproductive ability, which might make the title concise. So, ‘reproductive age’ can be deleted. Additionally, the author line may be revised as 3 merging the numbers 3,4 are the same organization, ‘CARE Bangladesh’.
2. In abstract:

- The author mentioned ’12 IDIs’, ‘Two FGDs’, and ‘Two KIIs’. 12 may be write into words. Also write the age range into a bracket that may be written as ‘adolescent girls aged 15-19’, avoiding the bracket.

- KIIs with one traditional birth attendant and one local pharmacist. As the study was conducted in an urban slum, there may be chances to find a trained birth attendant along with a traditional one. Because, NGOs are implementing many maternal health projects for the slum dwellers in parallel with governments. Inclusion of trained birth attendants by interviewing may add more or new reflections and dimensions of data and strengthen the results.

- Further, ‘local pharmacist’ is not an appropriate term in the context of Bangladesh, as they are already identified as ‘drug sellers’ considering their academic background and training. See (Ahmed SM, Naher N, Hossain T, Rawal, LB. Exploring the status of retail private drug shops in Bangladesh and action points for developing an accredited drug shop model: a facility based cross-sectional study. J Pharm Policy Pract. 2017).

-Results section, started with the recommendations like ‘adolescents need professional healthcare’. It’s better to start with the maternal health information sources and a trusted one. Therefore, the existing social norms need to describe how they ‘affect’ maternal health of adolescent girls and why these social norms are being influences. Delete the word ‘impact’ as your study observed not long term results.

- In conclusion, the author should be aware of using judgmental decisions like ‘without……information, no girl or woman’ would be able to make crucial decisions…. Health. Please revise.

3. Introduction section, the first sentence statistics were outdated. You can find the updated WHO Maternal Mortality: Key Facts data using the link <https://www.who.int/news-room/fact-sheets/detail/maternal-mortality>. In the line 6 of this section, the author mentioned that ‘Bangladesh has not been able to eliminate maternal mortality’, is it possible? Better to write ‘reduce’ the number following the WHO standard or like wealthier countries. However, the author summarizes the literature on maternal mortality and maternal health in global and Bangladesh contexts, health consequence and increasing risks among the adolescent. In doing so, it would be useful to further discuss the healthcare practices/facilities, especially for the adolescent group with maternal health information and behavior of Bangladesh, the role of healthcare providers, family members before the aim of the study in the introduction section. As your results already explored and you include this in your discussion part of the elder family member involvement issues in relying for health information. In the fourth para, fifth line from last, the author describe ‘women in making educated decisions…’ , could you explain it. The next line, ‘we found no studies on health information-seeking….is also judgmental. Suggesting write ‘paucity’ or ‘limited’ studies or others. In the same line the author contextualized the issue well describing slum population which citation is outdated (Khatun F. et al. 2012). The author can review the World Bank data, and even can cite Hasan MZ, Hasan AMR, Rabbani AG, Selim MA, Mahmood SS. 2022. Knowledge, attitude, and practice of Bangladeshi urban slum dwellers…… PLOS Global Public Health that identified “Around 38% (62.5 million) of the total population of this country live in urban areas and about half of them live in slums.” Please try to cite upto 2020 except classic references.
4. Methods could be better justified. This topic is likely to have significant social acceptability bias, which needs to be acknowledged. Specifically-

- The author need to contextualize the urban slum and then ‘Sattola’ describing the situation of slum, and its population in more details in the study site section.
- I believe this may be a typing error as it took ‘one month’ in 2.1 section and then ‘two months’ in the section 2.2.
- The sample size was not justified scientifically. The author could follow the data saturation principles for data collection is required here to fit in more within the qualitative paradigm. Better to include one reference here on data saturation related past/current article.
- Since the author recruited 12 married and unmarried adolescent girls, TBA and pharmacist (drug seller), it is worth mentioning the participants’ inclusion and exclusion criteria. Moreover, authors could discuss the challenges including attitudinal dimension that the team faced during data collection. Recruiting trained birth attendant and elder family member is already suggested in abstract section.
- Data collection procedures were not described well. Author could describe the research team academic background, training and experiences, interview guidelines (semi-structured or others), guidelines developing procedures, field test, interview settings (slums dwellers shared room, present about the ‘place' conducted interviews), recordings, participation type (volunteer or with pay)…. I suggest to follow or maximize the COREQ 32 items and may cited (<https://www.ncbi.nlm.nih.gov/books/NBK554122/>) this or any other related article.
- This is unclear what did the ‘deductive code list’ entail? As the study design is qualitative, why authors followed the deductive way is conflicting. Please revise this or explain.
- Thematic analysis procedures need to be explain more and need cited. Second paragraph of data analysis, author mentioned ‘themes were developed and recording was done…. understanding’ is unclear. Can revise following a reference. This could be more reflection on this and on the strengths (arguments, justifications for why the author use) which method brings to the analysis. In addition, ‘to confirm the accuracy…. and ‘cross-matched’ is overlap with triangulation, can revise.
- In ethical issues, the author mentioned followed …. ‘high priorities’.. need to explain how. ‘All of the participants gave their written consent’ need to revise. As the study participants were the garment workers and sat in for interview on their day off, please emphasize and highlight on the interview time, compensation, privacy and confidentiality (as they live in tiny and shared rooms) of the study participants and their shared experiences that ensured in the entire study. Because, author already described the hectic office hours of the participants and day off may their priority to get adequate rest. ‘One ethical specialist’ was recruited in the team, what was his/her roles. The author may revise the sentence … ‘destroyed data’ as the manuscript is not published yet and might need go back thorough data again.

5. The results are interesting but not line with study aim completely. However, it requires some reorganization of the results are following-

3.1 In theme 1, the author mentioned ‘maternal health information needs for adolescent girls’ is kind of assessment but aim of study was to ‘understand the maternal health information-seeking behavior and then primary goal was assess….., the author can revise the result section following the aim of study. Like, health information sources, trusted sources, social norms that influences the behavior, influences of health information seeking behavior, barriers/challenges in seeking behavior, and then needs of health information.

3.3 Moreover, I would ask to include interview number in the quotes.

6. **Discussion**: Suggesting knowledge is ‘accurate’ isn't very helpful with a qualitative study with such a small sample (and with no additional data, e.g. observations etc), but instead you can reflect on how knowledge is articulated or, as you do in the paper, discussing the role of the family in supporting to provide right information. Following the current literatures in introduction and reorganize the results section, the discussion need to be improved.
7. Conclusion: The first lines in the conclusion do not give any information that was intended to be explored in the study. In conclusion, readers want to see key findings interlinked with the social norms and health information seeking behavior that are based on empirical evidence, and then link those to policy recommendations.
