# Supplementary material for: Social norms and maternal health information-seeking behavior among adolescent girls: A qualitative study in a slum of Bangladesh
Source: PLoS One. 2024 Dec 6;19(12):e0315002. doi: 10.1371/journal.pone.0315002 (PMC11623481; doi:10.1371/journal.pone.0315002)
Supplement: Attachment — Submitted filename: Rebuttal Letter_Point by Point Response_PONE-D-23-34519R1.docx [file pone.0315002.s004.docx]

October 11, 2024

Ranjit Kumar Dehury

Academic Editor

PLOS ONE

Manuscript Number: PONE-D-23-34519R1

Title: Social norms and maternal health information-seeking behavior among adolescent girls: A qualitative study in a slum of Bangladesh

Dear Editor,

Thank you for allowing us to resubmit the revised manuscript. We appreciate the time and effort that you and the reviewers have dedicated to providing us with your valuable feedback on our manuscript. We are truly grateful to the reviewers for their insightful comments on our paper. We believe these suggestions will help improve the quality of the manuscript. We have been able to incorporate changes to reflect all the suggestions provided by the reviewers. We have used track changes option within the manuscript to highlight the changes.

Here is the point by point responses to the reviewers’ comments and concerns:

**Response to Reviewer #2:**

**Reviewer point #1:** The authors addressed all my comments. In the rebuttal table, they also explained how they addressed my comments. I am satisfied with their current version of the work. From my side, this version of work is quite fine.

**Author response #1:**

*Thank you very much for your positive feedback. We are delighted to hear that you found our manuscript well-conducted. We appreciate your hardwork to review our manuscript.*

**Response to Reviewer #3:**

**Reviewer point #1:** The title could be revised. ‘Adolescent girls of reproductive age’ is likely unnecessary, as adolescence represents the onset of reproductive ability, which might make the title concise. So, ‘reproductive age’ can be deleted. Additionally, the author line may be revised as 3 merging the numbers 3,4 are the same organization, ‘CARE Bangladesh’.

**Author response #1:**

*Thank you for catching this. We have removed these two words from the revised title. Author line is also revised. (page #1).*

**Reviewer point #2:** In abstract:

- The author mentioned ’12 IDIs’, ‘Two FGDs’, and ‘Two KIIs’. 12 may be write into words. Also write the age range into a bracket that may be written as ‘adolescent girls aged 15-19’, avoiding the bracket.

- KIIs with one traditional birth attendant and one local pharmacist. As the study was conducted in an urban slum, there may be chances to find a trained birth attendant along with a traditional one. Because, NGOs are implementing many maternal health projects for the slum dwellers in parallel with governments. Inclusion of trained birth attendants by interviewing may add more or new reflections and dimensions of data and strengthen the results.

- Further, ‘local pharmacist’ is not an appropriate term in the context of Bangladesh, as they are already identified as ‘drug sellers’ considering their academic background and training. See (Ahmed SM, Naher N, Hossain T, Rawal, LB. Exploring the status of retail private drug shops in Bangladesh and action points for developing an accredited drug shop model: a facility based cross-sectional study. J Pharm Policy Pract. 2017).

-Results section, started with the recommendations like ‘adolescents need professional healthcare’. It’s better to start with the maternal health information sources and a trusted one. Therefore, the existing social norms need to describe how they ‘affect’ maternal health of adolescent girls and why these social norms are being influences. Delete the word ‘impact’ as your study observed not long term results.

- In conclusion, the author should be aware of using judgmental decisions like ‘without……information, no girl or woman’ would be able to make crucial decisions…. Health. Please revise.

**Author response #2:**

*Thank you for your valuable suggestion.*

- *We have changed all the participants’s number in numerical form. Also, ‘adolescent girls aged 15-19’* *were changed from ‘adolescent girls (15-19 years old)’. (page #2)*
- *Your concern with KIIs is very much valid. We agree that another KII with trained birth attendant would give better understanding over the studied topic. However, as the participant ‘traditional birth attendant’ were trained by a non-government organization, we did not search for another one. For further clarification, we are adding a small description about the traditional birth attendant in the methodology section*
- *Thanks for suggesting the appropriate term. We have changed the term, accordingly, all aver the manuscript*
- *Result of the Abstract has been written accordingly. (page #2)*
- *Judgemental words were removed, accordingly*

**Reviewer point #3:** Introduction section, the first sentence statistics were outdated. You can find the updated WHO Maternal Mortality: Key Facts data using the link <https://www.who.int/news-room/fact-sheets/detail/maternal-mortality>. In the line 6 of this section, the author mentioned that ‘Bangladesh has not been able to eliminate maternal mortality’, is it possible? Better to write ‘reduce’ the number following the WHO standard or like wealthier countries. However, the author summarizes the literature on maternal mortality and maternal health in global and Bangladesh contexts, health consequence and increasing risks among the adolescent. In doing so, it would be useful to further discuss the healthcare practices/facilities, especially for the adolescent group with maternal health information and behavior of Bangladesh, the role of healthcare providers, family members before the aim of the study in the introduction section. As your results already explored and you include this in your discussion part of the elder family member involvement issues in relying for health information. In the fourth para, fifth line from last, the author describe ‘women in making educated decisions…’ , could you explain it. The next line, ‘we found no studies on health information-seeking….is also judgmental. Suggesting write ‘paucity’ or ‘limited’ studies or others. In the same line the author contextualized the issue well describing slum population which citation is outdated (Khatun F. et al. 2012). The author can review the World Bank data, and even can cite Hasan MZ, Hasan AMR, Rabbani AG, Selim MA, Mahmood SS. 2022. Knowledge, attitude, and practice of Bangladeshi urban slum dwellers…… PLOS Global Public Health that identified “Around 38% (62.5 million) of the total population of this country live in urban areas and about half of them live in slums.” Please try to cite upto 2020 except classic references.

**Author response #3:**

*Thank you for your insightful suggestion. The statistics of first sentence were updated. About the later suggestion: we agree that we need to put updated literature. However, I think there might be some technical issues that you reviewed the track change version or else. For instance, I had changed the following sentences/words: ‘Bangladesh has not been able to eliminate maternal mortality’, ‘women in making educated decisions…’ and ‘we found no studies on health information-seeking’ in the last revised submission. Regarding your suggestions on to ‘further discuss the healthcare practices/facilities, especially for the adolescent group with maternal health information and behavior of Bangladesh, the role of healthcare providers, family members before the aim of the study in the introduction section’ is addressed accordingly.We have added a paragraph regarding this. (page # 3 & 4)*

**Reviewer point #4:**  Methods could be better justified. This topic is likely to have significant social acceptability bias, which needs to be acknowledged. Specifically-
- The author need to contextualize the urban slum and then ‘Sattola’ describing the situation of slum, and its population in more details in the study site section.
- I believe this may be a typing error as it took ‘one month’ in 2.1 section and then ‘two months’ in the section 2.2.
- The sample size was not justified scientifically. The author could follow the data saturation principles for data collection is required here to fit in more within the qualitative paradigm. Better to include one reference here on data saturation related past/current article.
- Since the author recruited 12 married and unmarried adolescent girls, TBA and pharmacist (drug seller), it is worth mentioning the participants’ inclusion and exclusion criteria. Moreover, authors could discuss the challenges including attitudinal dimension that the team faced during data collection. Recruiting trained birth attendant and elder family member is already suggested in abstract section.
- Data collection procedures were not described well. Author could describe the research team academic background, training and experiences, interview guidelines (semi-structured or others), guidelines developing procedures, field test, interview settings (slums dwellers shared room, present about the ‘place' conducted interviews), recordings, participation type (volunteer or with pay)…. I suggest to follow or maximize the COREQ 32 items and may cited (<https://www.ncbi.nlm.nih.gov/books/NBK554122/>) this or any other related article.
- This is unclear what did the ‘deductive code list’ entail? As the study design is qualitative, why authors followed the deductive way is conflicting. Please revise this or explain.
- Thematic analysis procedures need to be explain more and need cited. Second paragraph of data analysis, author mentioned ‘themes were developed and recording was done…. understanding’ is unclear. Can revise following a reference. This could be more reflection on this and on the strengths (arguments, justifications for why the author use) which method brings to the analysis. In addition, ‘to confirm the accuracy…. and ‘cross-matched’ is overlap with triangulation, can revise.
- In ethical issues, the author mentioned followed …. ‘high priorities’.. need to explain how. ‘All of the participants gave their written consent’ need to revise. As the study participants were the garment workers and sat in for interview on their day off, please emphasize and highlight on the interview time, compensation, privacy and confidentiality (as they live in tiny and shared rooms) of the study participants and their shared experiences that ensured in the entire study. Because, author already described the hectic office hours of the participants and day off may their priority to get adequate rest. ‘One ethical specialist’ was recruited in the team, what was his/her roles. The author may revise the sentence … ‘destroyed data’ as the manuscript is not published yet and might need go back thorough data again.

**Author response #4:**

*Thanks for your valuable suggestions.*

- *We have added some description about urban slums of Bangladesh. The exact population were not found from any formal sources.*

- *In the revised version, I edited this typing error. It would be one month, for your reference. Moreover, in the revised version, I did not put any section numbers, such as, 2.1 or 2.1*

- *Yes. I totally agree with your concern that the sample size was not justified scientifically. Somehow, we missed that. Thanks for your comment. We have explained that in the* ***Sampling and Recruitment*** *section. (page #6-7)*

*- Thanks for the comment on clarifying inclusion and exclusion criteria. We have incorporated your concern accordingly. (page #6-7)*

*- Thanks very much for this suggestion. Data collection procedures are re-written and rearranged as per your concern. The background of the data collectors were written in* ***Sampling and Recruitment*** *section, earlier. Procedures of guidelines developing and field test experience are described in the section* ***Study Design.*** *The whole methodology were revised according to COREQ. Also, we have added a supplementary file for your reference.*

- This is unclear what did the ‘deductive code list’ entail? As the study design is qualitative, why authors followed the deductive way is conflicting. Please revise this or explain.

- *The concern for ‘deductive code list’ has been revised.*

*- Thematic analysis procedures have been cited. Other concerns have been addressed.*

*- We mentioned ‘high priorities’.. as it ensures to make no harm for the participant and maintain confidentiality as we promised them before interviewing. Then, there was a mistake about the consent. It would be oral consent. Information about participant’s compensation, privacy and confidentiality are added in the above sections. Explanation regarding ‘One ethical specialist’ are added. In addition, your concern are very much legit about the ‘destroyed data’. There was flaws. It was not written properly. Now, we have edited the issue. (page #9)*

**Reviewer point #5:** The results are interesting but not line with study aim completely. However, it requires some reorganization of the results are following-

3.1 In theme 1, the author mentioned ‘maternal health information needs for adolescent girls’ is kind of assessment but aim of study was to ‘understand the maternal health information-seeking behavior and then primary goal was assess….., the author can revise the result section following the aim of study. Like, health information sources, trusted sources, social norms that influences the behavior, influences of health information seeking behavior, barriers/challenges in seeking behavior, and then needs of health information.

3.3 Moreover, I would ask to include interview number in the quotes.

**Author response #5:**

*Thank you for your insightful comment.*

*3.1 In the revised version, I updated the foremost and specific objectives. It’s true that our major objective is to understand* *maternal health information seeking behaviour. However, we believe that it would be better understood if we understand the need, at first, and then, the barriers or, more specifically, the influences of social norms.(page #4) In addition, the thematic order has been revised.*

*3.3 Interview number in the quotes are added.*

**Reviewer point #6:** Discussion: Suggesting knowledge is ‘accurate’ isn't very helpful with a qualitative study with such a small sample (and with no additional data, e.g. observations etc), but instead you can reflect on how knowledge is articulated or, as you do in the paper, discussing the role of the family in supporting to provide right information. Following the current literatures in introduction and reorganize the results section, the discussion need to be improved.

**Author response # 6:**

*Thanks for your critical observation. We have changed such words like ‘accurate’. We have revised the discussion*

**Reviewer point #7:** Conclusion: The first lines in the conclusion do not give any information that was intended to be explored in the study. In conclusion, readers want to see key findings interlinked with the social norms and health information seeking behavior that are based on empirical evidence, and then link those to policy recommendations.

**Author response #7:**

*Thank you for your feedback. We have edited the conclusion accordingly. (page #24-25)*
